# Supplementary material for: Elastic strain-induced amorphization in high-entropy alloys
Source: Nat Commun. 2024 May 30;15:4599. doi: 10.1038/s41467-024-48619-0 (PMC11139900; doi:10.1038/s41467-024-48619-0)
Supplement: Supplementary file 1 — Supplementary Information [file 41467_2024_48619_MOESM1_ESM.pdf]

Supplementary Information

Elastic strain-induced amorphization in high-entropy alloys

Yeqiang Bu[1,2#], Yuan Wu[3,4#*], Zhifeng Lei[5], Xiaoyuan Yuan[3], Leqing Liu[3], Peng Wang[6], Xiongjun Liu[3], Honghui Wu[3], Jiabin Liu[1,7*], Hongtao Wang[1,2*], R. O. Ritchie[8], Zhaoping Lu[3*], Wei Yang[1]

[1]Center for X-mechanics, School of Aeronautics and Astronautics, Zhejiang University, Hangzhou 310027, China

[2]ZJU-Hangzhou Global Scientific and Technological Innovation Center, Zhejiang University, Hangzhou 310027, China

[3]Beijing Advanced Innovation Center for Materials Genome Engineering, State Key Laboratory for Advanced Metals and Materials, University of Science and Technology Beijing, Beijing 100083, China

[4] Institute for Materials Intelligent Technology, Liaoning Academy of Materials, Shenyang, 110010, China

[5]College of Materials Science and Engineering, Hunan University, Changsha 410082, China

[6]Shanghai Key Laboratory of Mechanics in Energy Engineering, Shanghai Institute of Applied Mathematics and Mechanics, School of Mechanics and Engineering Science, Shanghai University, Shanghai 200444, China

[7] State Key Laboratory of Silicon and Advanced Semiconductor Materials, School of Materials Science and Engineering, Zhejiang University, Hangzhou 310027, China

[8]Department of Materials Science and Engineering, University of California, Berkeley, CA 94720, USA

[#]These authors contributed equally to the work.

To whom correspondence should be addressed. E-mails: wuyuan@ustb.edu.cn *(Yuan Wu),* liujiabin@zju.edu.cn *(Jiabin Liu),* htw@zju.edu.cn *(Hongtao Wang) and* luzp@ustb.edu.cn *(Zhaoping Lu)*

This PDF file includes:

Supplementary discussion 1 -3

Supplementary Table 1

Supplementary Figures 1-18

Supplementary References

Supplementary discussion

1. Evaluation for the effects of electron irradiation

It should be noted that electron irradiation may also artificially induce amorphization. Here, the effects of electron irradiation were fully evaluated through “knock on” and “heating” effects [1,2]. To evaluate the "knock-on" effects of electron irradiation on the nanoscale HEA samples, we exposed a nanoscale TiHfZrNb sample to an electron beam for 30 minutes. Subsequent examination of this sample revealed that the shape and location of crystal-amorphous interface, crystal orientation and lattice spacing in the TEM pictures and the corresponding FTT image (Fig. S3) remained unchanged after irradiation, indicating that the incident beam did not cause a “knock on” effect during this exposure period. As for any “heating” effect, a theoretical estimate on the temperature rise induced by electron irradiation is shown in following formulas [3,4]:

$$\Delta T = \frac{I}{4\pi K e}\left(\frac{\Delta E}{d}\right)\left(1 + 2\ln\frac{b}{r_0}\right) \quad (1)$$

$$-\frac{\Delta E}{d} = \frac{2\pi Z\rho(e^2/4\pi\varepsilon_0)^2}{mv^2}\left\{\ln\left[\frac{E(E+mc^2)^2\beta^2}{2I_e^2mc^2}\right] + (1-\beta^2) - (1-\sqrt{1-\beta^2}+\beta^2)\ln 2 + \frac{1}{8}(1-\sqrt{1-\beta^2})^2\right\}$$

(2)

where K is the thermal conductivity of the sample, I is the beam current, ΔE is the total energy loss per electron in a sample of thickness d, b is the radius of the heat sink, and r_0 is the beam radius, Z is the atomic number of the samples, ρ is the atomic density, ε_0 is the vacuum dielectric constant, m is the electron rest mass, v is the electron velocity, c is the speed of light, E is the electron energy, I_e is the average excitation energy of electrons in the target, and $\beta = v/c$. Given $K = 7.955$ W(m·K)$^{-1}$, $I = 3$ nA, $b = 1.5$ mm, $\rho = 2.58\times10^{28}$ m^{-3}, $r_0 = 200$ nm, $Z = 43.75$, $e = 1.6\times10^{-19}$ C, $\varepsilon_0 = 8.85\times10^{-12}$ F·m^{-1}, $m = 9.3\times10^{-31}$ kg, $v = 2.0837\times10^{8}$ m·s^{-1}, $c = 3.0\times10^{8}$ m·s^{-1}, $E = 200$ keV, $I_e = 385$ eV, we calculated the temperature rise (ΔT) to be 0.45 K, which can be neglected. However, it is possible that other artificial effects could be caused by the synergy of the electron

beam and applied stress. Comparing the pre- and post-mortem high-resolution images, strain-induced amorphization is still observed in the TiHfZrNb sample stretched in the beam blank condition (Fig. S4). Accordingly we conclude that the effects of electron irradiation on the deformation behavior of the samples can be neglected.

2. Elastic instability via dislocation-related behaviors in bulk TiHfZrNb

The post-mortem microstructure characterization of bulk TiHfZrNb samples after tensile test has been closely performed in our previous work [5]. The TEM and XRD results show that dislocation slip mediates the deformation of bulk TiHfZrNb, while elastic strain-induced amorphization is absent. The *in situ* TEM tensile test of the TiHfZrNb foil with submicron thickness has also been performed in our previous work [5], where extensive dislocation nucleation and propagation were observed.

We investigated the post-mortem microstructure of the bulk TiHfZrNb samples heavily cool-rolled with a reduction ratio of 90%. Several band-like structures were observed, as shown in Fig. S6a. Figure S6b is the corresponding SAED pattern, where a diffuse diffraction ring can be clearly recognized, indicating occurrence of the localized amorphization in those bands. Such localized amorphization event was further confirmed by high-resolution TEM images (Figs. S6c and d). Nevertheless, it is critical to point out that the formation mechanism of those amorphous bands is totally different from that in our nanoscale samples. We carefully characterized the microstructure of the 30% rolled bulk TiHfZrNb alloy, as shown in Fig. S7, where only a high density of dislocations was observed without any sign of amorphous structure. Therefore, the low elastic strain limit of bulk alloys (usually less than 1%) would lead to the prevalent dislocation generation and the resultant defect accumulation amorphization, similar to what had been reported in extremely deformed bulk CrMnFeCoNi HEAs [6]. Such defect accumulation process inhibits the elastic strain-induced amorphization in bulk TiHfZrNb HEA, resulting in a completely different responses to external stress.

3. Energy landscape of elastic strain-induced amorphization

The energy landscape of elastic strain-induced amorphization is studied using First Principles (Density Functional Theory) simulations. The energy of the amorphous and crystalline structures, as well as the system energy evolution of TiHfZrNb with elastic strain, was calculated. The amorphous configuration was obtained by quenching the liquid TiHfZrNb from 3000 K to 0 K at an infinite cooling rate (Fig. S13a). The energy of the amorphous TiHfZrNb was calculated as -8.981 eV $atom^{-1}$. The energy of the relaxed atomic configuration of crystalline TiHfZrNb (Fig. S13b) was calculated as -9.037 eV $atom^{-1}$, which is lower than that of the amorphous state. As elastic strain increases, the energy of the TiHfZrNb lattice gradually increases (Fig. S13d) and reaches -8.975 eV $atom^{-1}$ at an elastic strain of 10% (Fig. S13c), which exceeds that of the amorphous state and indicates that the highly tension-loaded samples are unstable compared to the amorphous state. These results suggest that the energy barrier between the crystal and its amorphous counterpart can be overcome by storing elastic strain energy through a large elastic strain (~10%).

Supplementary Table 1. The elastic instability mechanisms of alloys with various number of principal elements

Alloys	elastic instability mechanism
TiHfZrNbTa	Elastic strain-induced amorphization
TiHfZrNb	Elastic strain-induced amorphization
TaHfZrNb	Elastic strain-induced amorphization
TiZrNb	Elastic strain-induced amorphization
HfZrNb	Elastic strain-induced amorphization
TiHfNb	Elastic strain-induced amorphization
TiNb	Crystalline phase transformation
ZrNb	Crystalline phase transformation
TiZr	Partial dislocations
Nb [11]	Crystalline phase transformation, twinning and dislocations

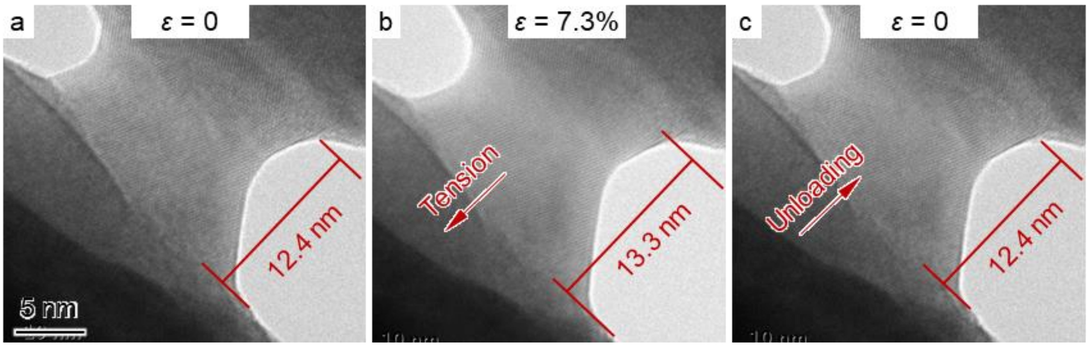

Supplementary Figure 1 | Loading-unloading during tension of a TiHfZrNb sample. **a**, The initial TiHfZrNb sample; **b**, The TiHfZrNb sample is elastically stretched to 7.3% without any inelastic relaxation; **c**, The TiHfZrNb sample recovers its initial shape after complete unloading.

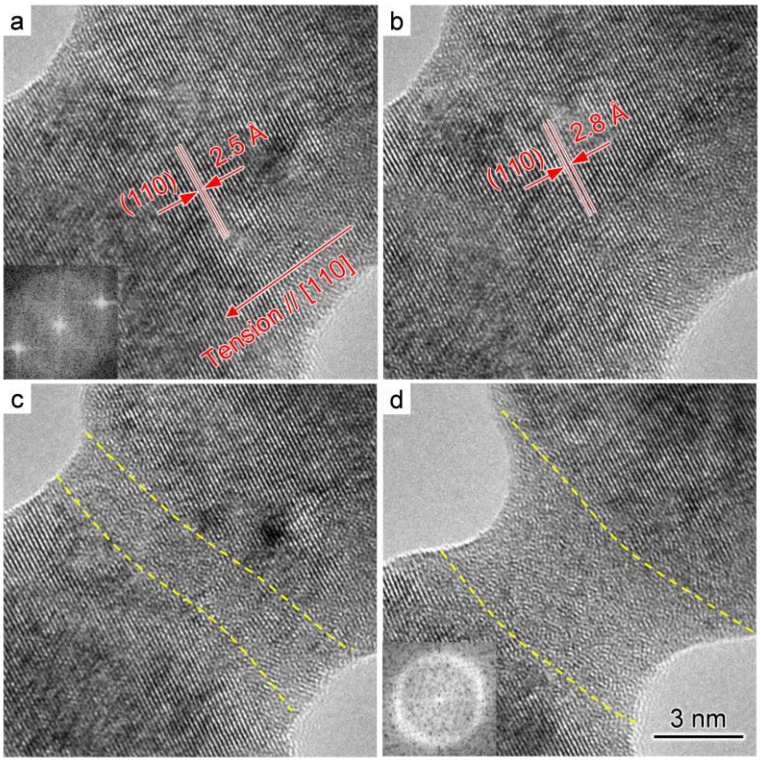

Supplementary Figure 2 | Another example of the amorphization in [110] tension-loaded TiHfZrNb sample. a, A crystalline HEA sample was loaded in tension around [110]. **b**, The critical lattice with the elastic strain of 10% (determined by the (110) lattice spacing change) prior to the occurrence of amorphization. **c**, A segment of amorphous structure (outlined by a pair of yellow dashed lines) formed in the area with severe elastic deformation. **d**, The amorphous segment propagates as further tensile load applies. The insets in **a** and **d** are corresponding FFT pattern, which show the crystalline and amorphous nature of the sample before and after tensile test, respectively.

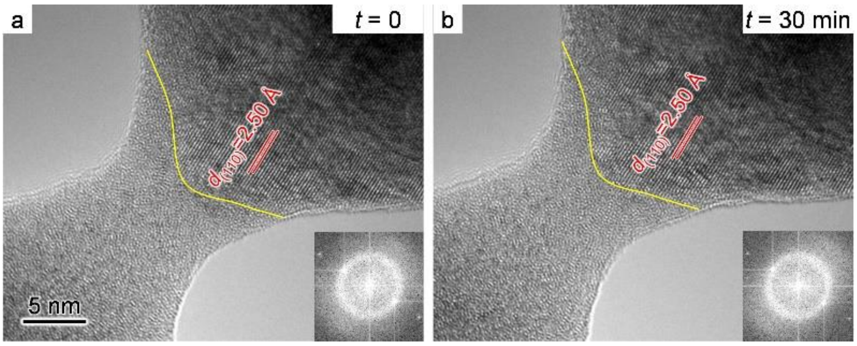

Supplementary Figure 3 | A nanoscale HEA sample was exposed to electron irradiation for 30 min. No structural change can be detected (**a**) before and (**b**) after irradiation. The crystal-amorphous interface was outlined by the yellow lines, and the insets are corresponding FFT images. Insets in **a** and **b** are corresponding FFT pattern.

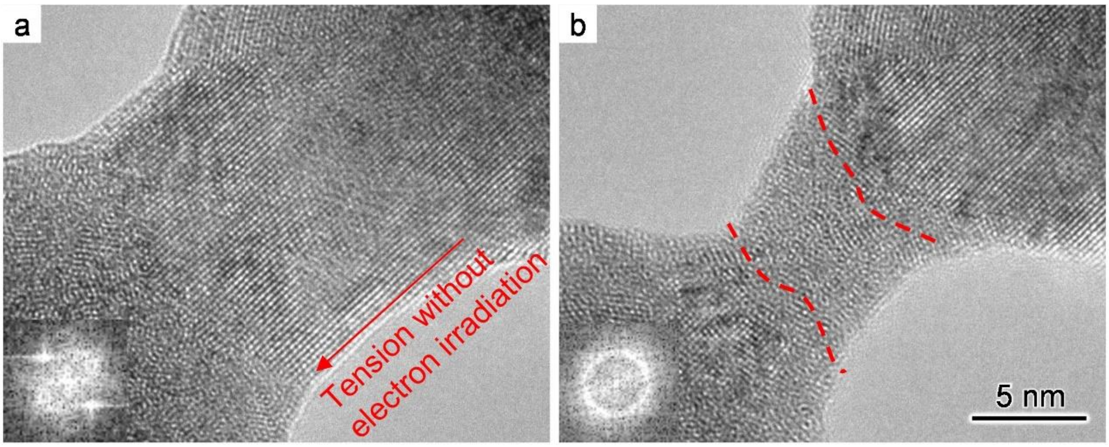

Supplementary Figure 4 | A TiHfZrNb HEA sample was stretched with electron beam switched off. a, The initial HEA lattice. **b**, A strain-induced amorphous segment (outlined by a pair of red dashed lines) was still produced when the beam was switched off. Insets are the corresponding FFT pattern, showing the crystalline and amorphous nature of the sample before and after tensile test, respectively.

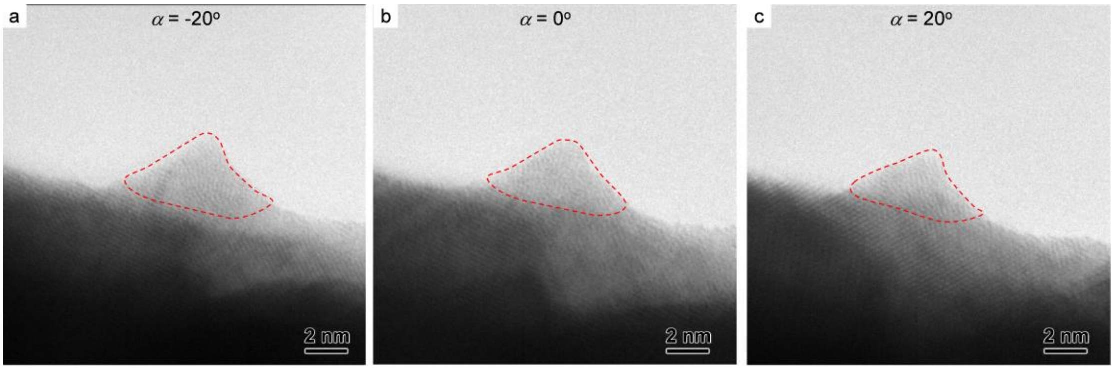

Supplementary Figure 5 | High-resolution TEM images viewed at different tilting angles. (a) α = -20°, (b) α = 0° and (c) α = 20° of a TiHfZrNb sample containing amorphous structure (outlined by the dashed red line) generated by elastic strain-induced amorphization.

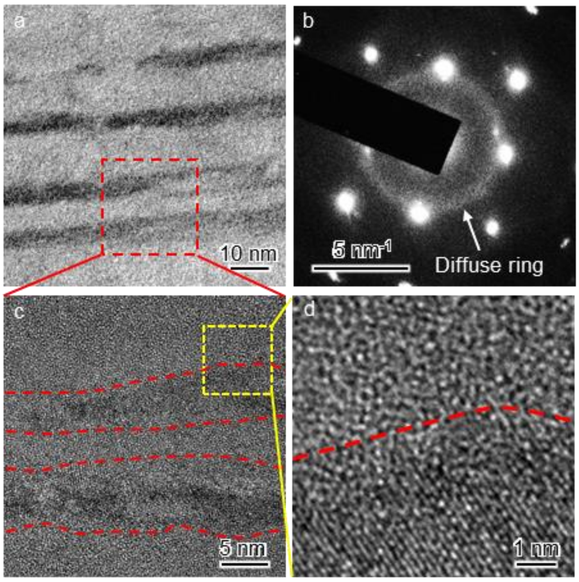

Supplementary Figure 6 | Amorphous band in 90% rolled bulk TiHfZrNb sample. **a,** Bright-field TEM image for the band-like structure in 90% rolled bulk TiHfZrNb sample. **b**, The corresponding selected area diffraction pattern. **c**, High-resolution TEM image shows the amorphous and crystalline bands. **d**, High-resolution TEM image shows the interface of amorphous structure and crystal.

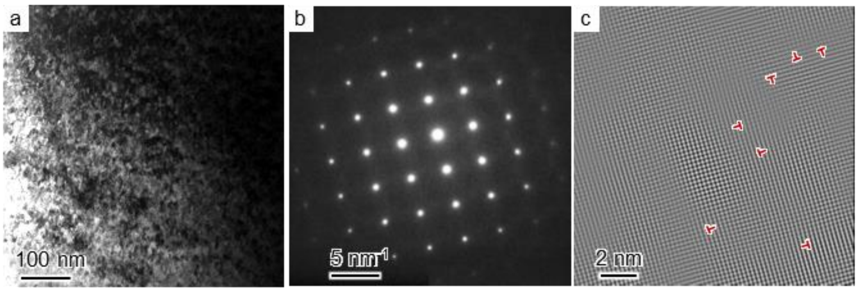

Supplementary Figure 7 | High density of dislocations in 30% rolled bulk TiHfZrNb sample. a, Bright-field TEM image for the microstructure of 30% rolled bulk TiHfZrNb sample. **b**, Corresponding selected area diffraction pattern. **c,** High-resolution image to reveal the dislocation cores.

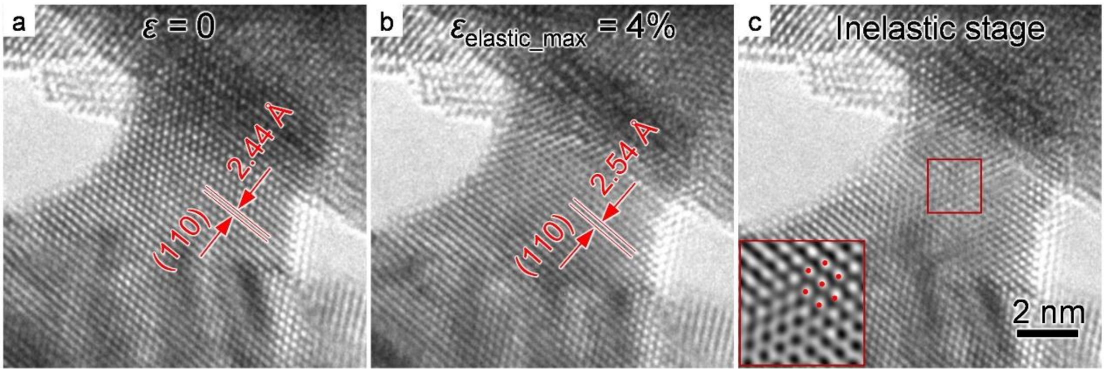

Supplementary Figure 8 | *In situ* high-resolution TEM photographs reveal the critical configuration of nanoscale ZrNb before crystalline transformation occurs. **a**, Initial nanoscale ZrNb lattice; **b**, Elastically deformed lattice at a critical state; **c**, ZrNb lattice containing FCC lattice.

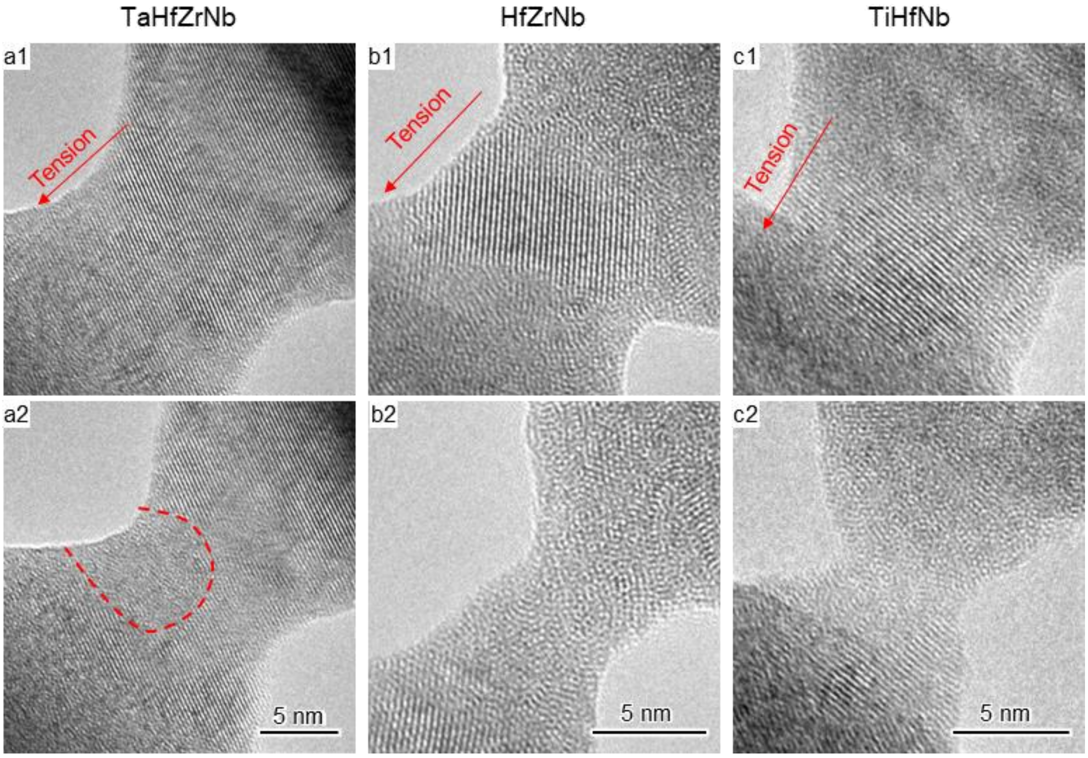

Supplementary Figure 9 | Elastic strain-induced amorphization in samples of the quaternary alloy TaHfZrNb (a1, before tension, a2, after tension), **ternary alloys HfZrNb** (b1, before tension, b2, after tension) **and TiHfNb** (c1, before tension, c2, after tension)**, respectively.**

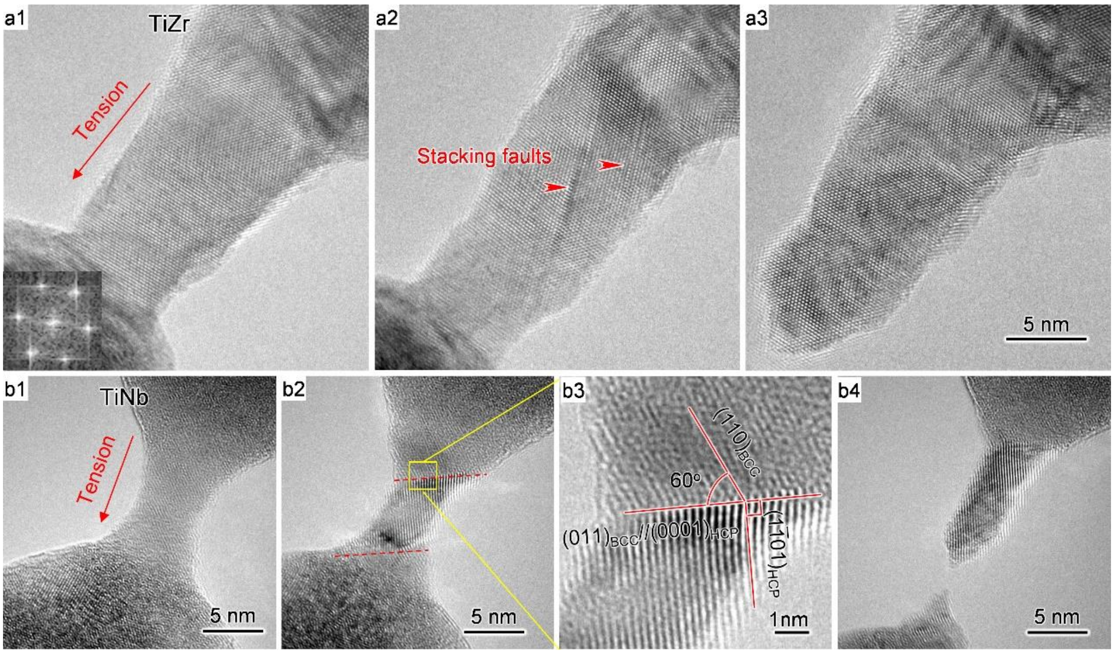

Supplementary Figure 10 | Elastic instability in binary alloy samples is dominated by dislocations and crystalline phase transformation. a1-3, The elastic instability of TiZr sample with a hexagonal close packed (HCP) lattice is dominated by partial dislocations without elastic strain-induced amorphization. **b1-4**, A transformation from the BCC to HCP lattice was detected in the TiNb sample without elastic strain-induced amorphization. **b3** shows the orientation relationship between BCC and the product HCP lattice.

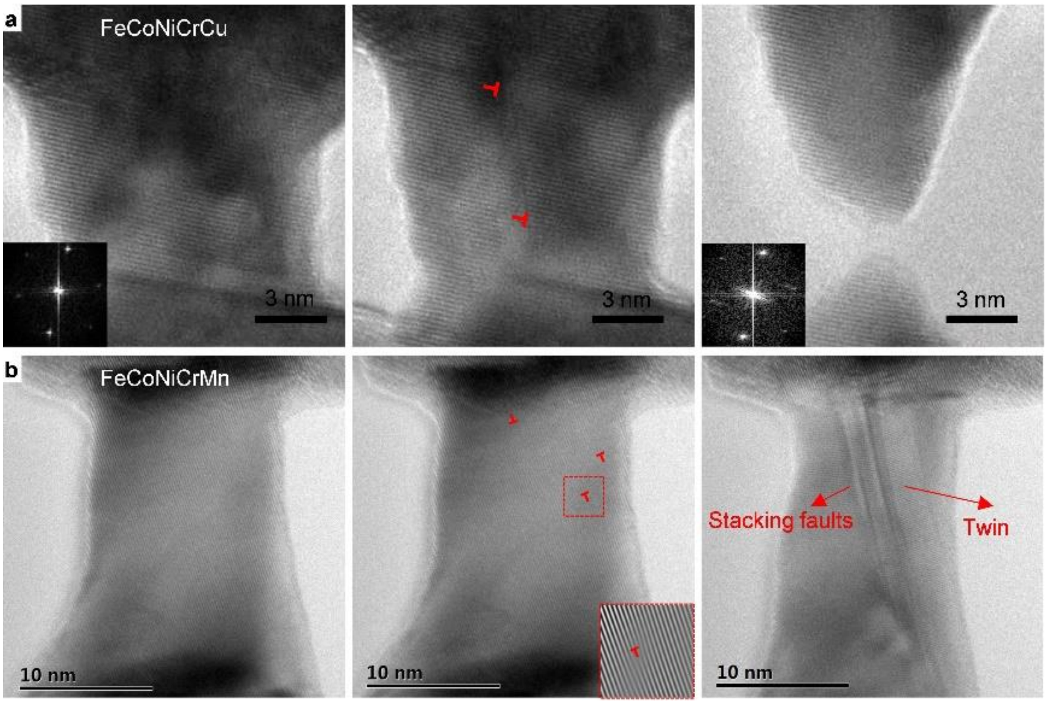

Supplementary Figure 11 | Dislocations mediated elastic instability in nanoscale quinary FCC-structured (a) FeCoNiCrCu and **(b)** FeCoNiCrMn. Insets in **a** are corresponding FFT pattern, showing the crystalline nature of the sample before and after tensile test. Inset in **b** is an enlarged view of the dashed square area.

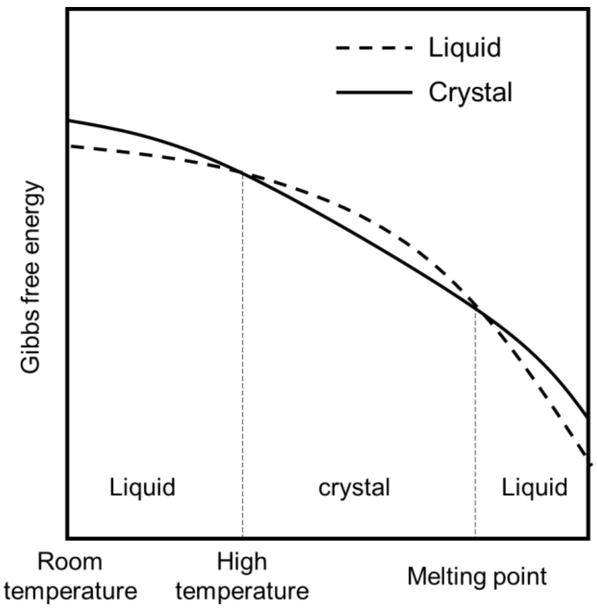

Supplementary Figure 12 | Schematic image show the variation of Gibbs free energy with temperature when low-temperature amorphization occurs in a crystal. It is remarkable that the crystal involved must have a higher entropy ($S = -(\partial G/\partial T)$, corresponding to the slope of the Gibbs free energy curves) than the liquid phase (i.e., amorphous structure) at low temperature. The coordination number in liquids and glasses, 11 -12, is close to 12 in close-packed FCC crystals, but is significantly higher than 8 in non-close-packed BCC crystals. BCC crystals will generally have a higher vibrational entropy than close-packed FCC crystals and amorphous structure, which favors the amorphization at low-temperature.

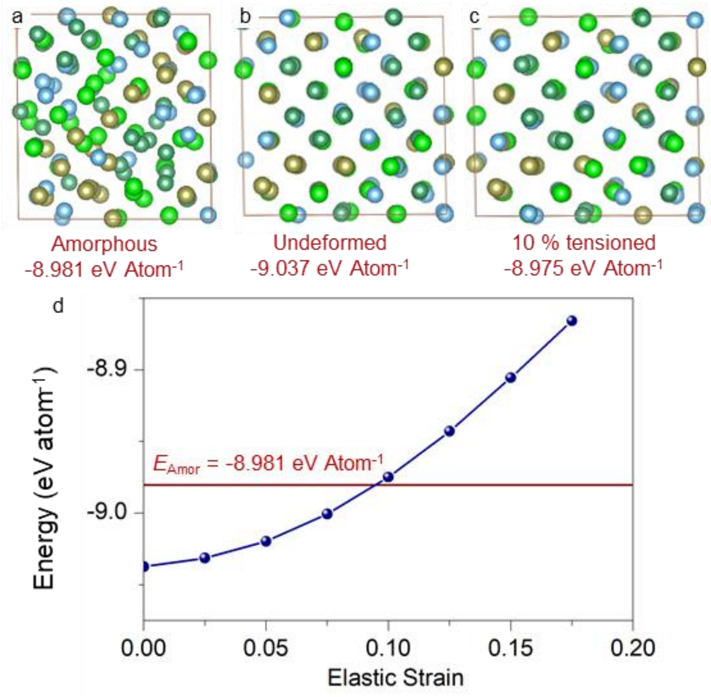

Supplementary Figure 13 | Energy storage through elastic deformation. a-c, Atomic configurations of amorphous TiHfZrNb, undeformed crystalline TiHfZrNb and loaded to a tensile strain of 10% in TiHfZrNb, respectively. **d**, The energy and stress of TiHfZrNb lattice increases with elastic strain; the black line represents the energy of the amorphous configuration of TiHfZrNb in **a**.

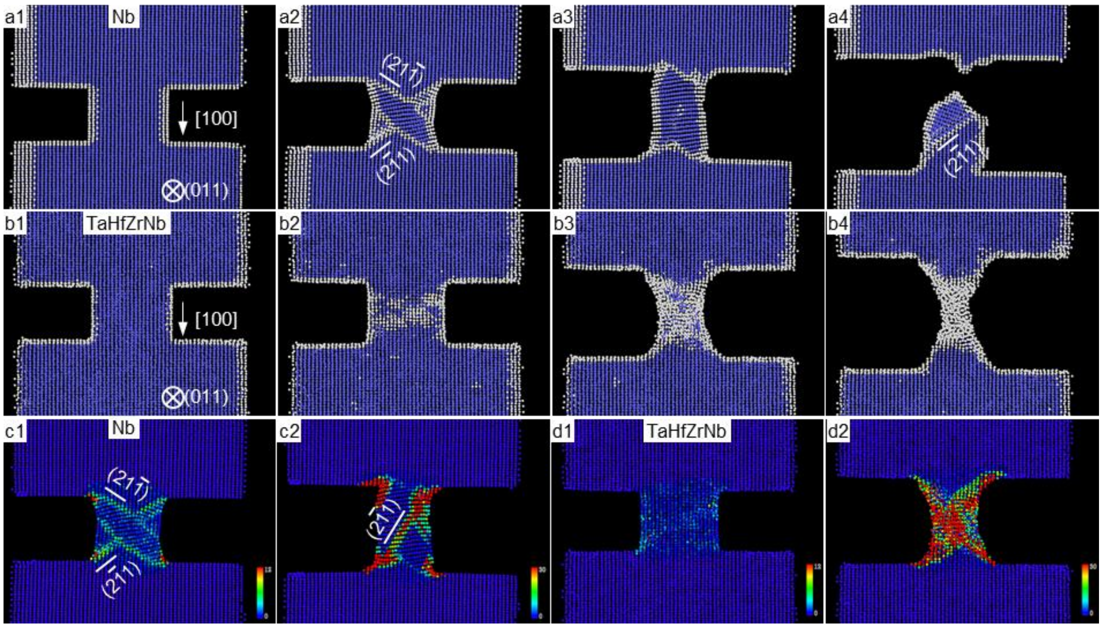

Supplementary Figure 14 | Molecular dynamic simulations for the lattice evolution on (011) plane with tensile strain in nanoscale elemental Nb (a1-a4) and TaHfZrNb HEA (b1-b4). Two samples have the same initial configuration (**a1, b1**). Dislocation-mediated elastic instability is found in the nanoscale elemental Nb after reaching the elastic strain limit (**a**2). **a3-a4** show the plastic deformation of Nb is dominated by dislocation slip; the nanoscale Nb retains an ordered lattice even after fracture occurs. **b2** shows that several disordered regions emerge in TaHfZrNb after the elastic limit is reached, which indicates that the elastic instability in TaHfZrNb is mediated by elastic strain-induced amorphization. **b3-b4** show the disordered regions coarsened during further plastic deformation. **c1** and **c2** show the non-affine atomic displacements mapping of Nb, corresponding to **a2** and **a3**. **d1** and **d2** show the non-affine atomic displacements mapping of TaHfZrNb, corresponding to **b2** and **b3**. The observed experimental scenarios are well reproduced in MD simulations, which show that the mechanisms underpinning the elastic instability in elemental Nb and the quaternary TaHfZrNb are, respectively, dislocation slip and elastic strain-induced amorphization. Additionally, non-affine atomic displacement mapping of Nb at strains exceeding the elastic strain limit shows that the atomic displacements are focused on the $\{112\}$ slip plane, which corresponds to the glide of dislocations. However, the non-affine atomic

displacements in the quaternary TaHfZrNb are extensively distributed in an irregular manner at strains above the elastic strain limit of ~10%, which activates the elastic strain-induced amorphization phenomenon. Atoms in blue represent atoms in a BCC structure; those in silver white represent atoms in a disordered structure. Atoms in other colors represent the value of non-affine displacement scaling with the scale bar in (**c1, c2, d1** and **d2**). Both MD samples possess a circular cross-section. Affine displacement is a geometric transformation that linearly alters the position of atoms in space, reflecting the uniform movement of atoms along the slip plane facilitated by dislocation slipping. Conversely, non-affine displacement captures the disordered movement of atoms during amorphization. Additionally, our research focuses on the middle position of the sample, which is characterized by stress concentration and exhibits a circular cross-section in both experimental and simulated conditions. This midpoint is far away from the specimen ends where external loads were applied. As a result, we can confidently disregard any deviation in stress distribution associated with variations in the shape of the specimen ends, as illustrated by St. Venant's principle[7].

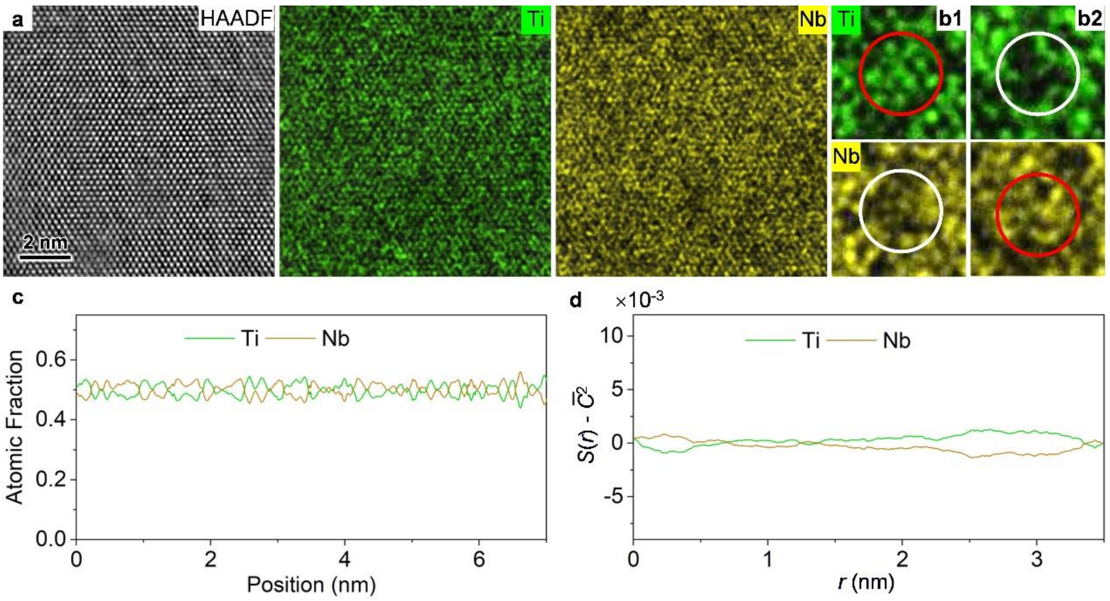

Supplementary Figure 15 | Atomic-scale element distribution analysis for binary TiZrNb alloys. **a**, HAADF-STEM image along the [111] zone axis, and corresponding atomically resolved EDS maps for individual elements of Ti and Nb. **b1-2,** Zoomed-in images captured from the local regions in **a**, compared with the various local chemical groups in ternary TiZrNb alloy, there are just two kinds of local groups in TiNb alloy, i.e., the Ti- and Nb-rich local groups. **c,** Line profiles representing the distribution of individual elements in $(1\bar{1}0)$ planes projected along the [111] direction. Compared with TiZrNb alloy, the variation of atomic fraction of individual elements is smaller. **d,** Plots of the pair correlation function of individual elements of TiNb alloy, which possess low and broad peaks for each element, indicating the elements distribution in TiNb alloy is random.

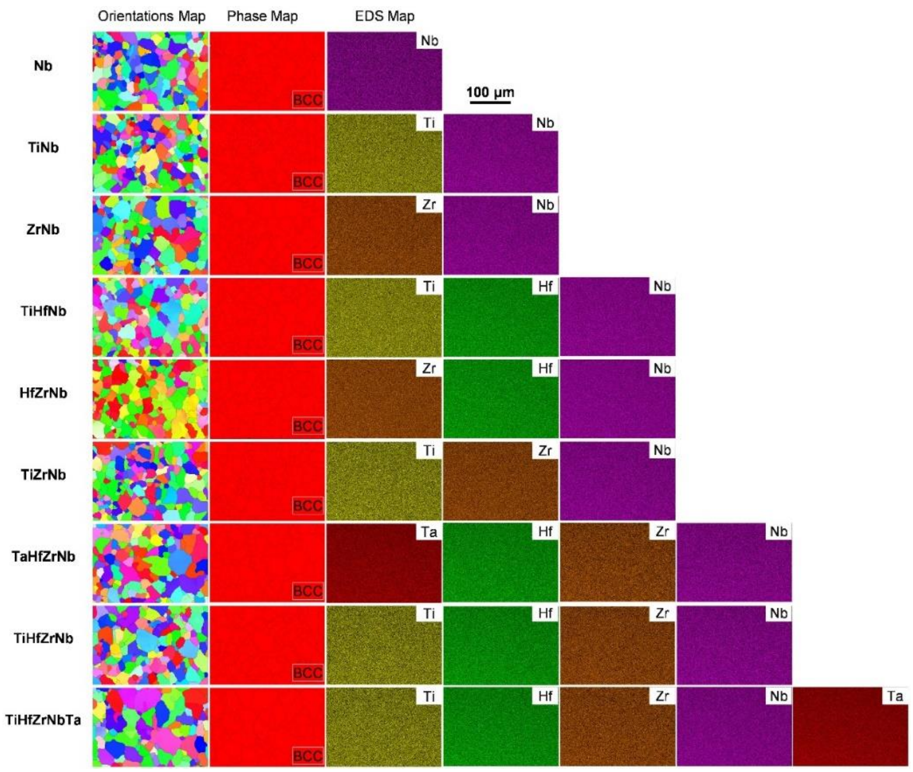

Supplementary Figure 16 | EBSD orientation map (the leftmost column)**, EBSD phase map** (second column from left) **and EDS maps of each constituent element** (the other images with element symbol on the upper right corner) **for the samples used in this study.**

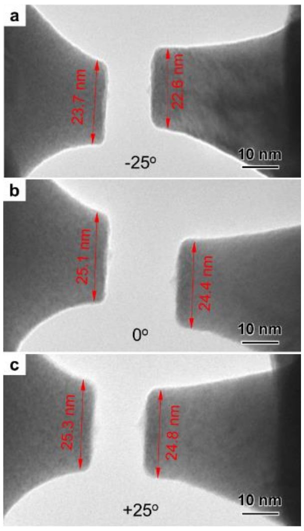

Supplementary Figure 17 | Cross-section of the nanoscale HEA sample observed by tilting the nanoscale sample along the *α* direction from -25° to +25°. Cross-section images of the nanoscale sample tilted at angles of (a) -25°, (b) 0° and (c) +25°. The diameter of the nanoscale sample changes little, which indicates that the cross section of the nanoscale HEA sample is nearly circular.

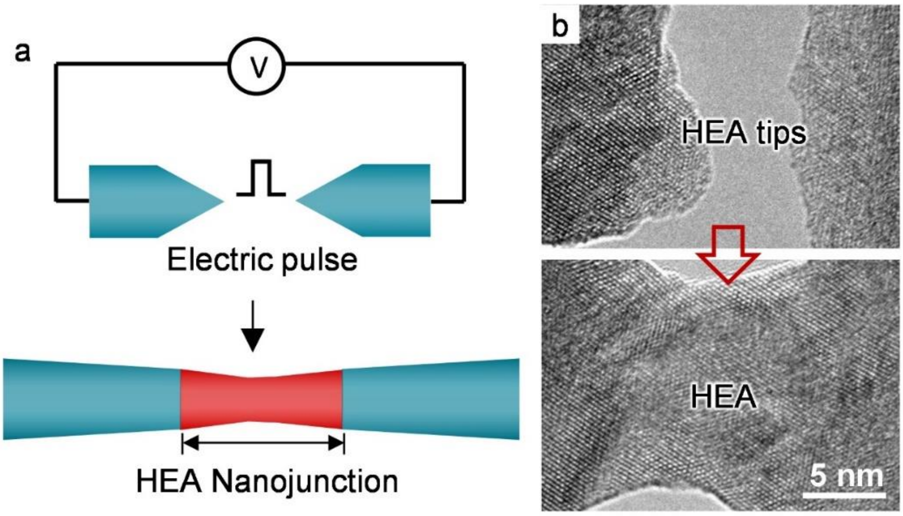

Supplementary Figure 18 | Preparation of the sample. a, A schematic illustration of *in situ* welding; **b**, A HEA sample prepared from HEA nano-tips by an electric pulse with duration time of 1 ms and voltage range of 0-2V.

Supplementary References

1 Dong, C. *et al.* Evolution of Pt clusters on graphene induced by electron irradiation. *J. Appl. Mech.* **80**, 040904 (2013).

2 Sun, J. *et al.* Liquid-like pseudoelasticity of sub-10-nm crystalline silver particles. *Nature Mater.* **13**, 1007-1012 (2014).

3 Fisher, S. On the temperature rise in electron irradiated foils. *Radiation Effects* **5**, 239-243 (1970).

4 Jencic, I., Bench, M., Robertson, I. & Kirk, M. Electron-beam-induced crystallization of isolated amorphous regions in Si, Ge, GaP, and GaAs. *J. Appl. Phys.* **78**, 974-982 (1995).

5 Bu, Y. *et al.* Local chemical fluctuation mediated ductility in body-centered-cubic high-entropy alloys. *Mater. Today* **46**, 28-34 (2021).

6 Zhao, S. *et al.* Amorphization in extreme deformation of the CrMnFeCoNi high-entropy alloy. *Sci. Adv.* **7**, eabb3108 (2021).

7 Craig, R., Taleff, E. M. Mechanics of materials. *John Wiley & Sons*, 2020.
