# Supplementary material for: Elastic strain-induced amorphization in high-entropy alloys
Source: Nat Commun. 2024 May 30;15:4599. doi: 10.1038/s41467-024-48619-0 (PMC11139900; doi:10.1038/s41467-024-48619-0)
Supplement: Supplementary file 3 — Description of Additional Supplementary Information [file 41467_2024_48619_MOESM3_ESM.pdf]

Caption for Supplementary Movie 1.

Supplementary Movie 1.

In situ process revealing the amorphization of nanoscale TiHfZrNb.
